# Molecular design of radiocopper-labelled Affibody molecules

**DOI:** 10.1038/s41598-018-24785-2

**Published:** 2018-04-25

**Authors:** Vladimir Tolmachev, Tove J. Grönroos, Cheng-Bin Yim, Javad Garousi, Ying Yue, Sebastian Grimm, Johan Rajander, Anna Perols, Merja Haaparanta-Solin, Olof Solin, Riccardo Ferdani, Anna Orlova, Carolyn J. Anderson, Amelie Eriksson Karlström

**Affiliations:** 1 Department of Immunology, Genetics and Pathology, Uppsala University, Uppsala, Sweden; 2 Turku PET Centre, University of Turku, Turku, Finland; 3 MediCity Research Laboratory, University of Turku, Turku, Finland; 4 Department of Oncology and Radiotherapy, Turku University Hospital, Turku, Finland; 5 Turku PET Centre, Åbo Akademi University, Turku, Finland; 6 Department of Protein Science, KTH Royal Institute of Technology, Stockholm, Sweden; 7 Department of Chemistry, University of Turku, Turku, Finland; 8 Washington University, St. Louis, MO USA; 9 Department of Medicinal Chemistry, Uppsala University, Uppsala, Sweden; 10 Departments of Medicine, Radiology, Bioengineering and Pharmacology & Chemical Biology, University of Pittsburgh, Pittsburgh, PA 15203 USA

## Abstract

The use of long-lived positron emitters ^64^Cu or ^61^Cu for labelling of Affibody molecules may improve breast cancer patients’ stratification for HER-targeted therapy. Previous animal studies have shown that the use of triaza chelators for ^64^Cu labelling of synthetic Affibody molecules is suboptimal. In this study, we tested a hypothesis that the use of cross-bridged chelator, CB-TE2A, in combination with Gly-Glu-Glu-Glu spacer for labelling of Affibody molecules with radiocopper would improve imaging contrast. CB-TE2A was coupled to the N-terminus of synthetic Affibody molecules extended either with a glycine (designation CB-TE2A-G-ZHER2:342) or Gly-Glu-Glu-Glu spacer (CB-TE2A-GEEE-ZHER2:342). Biodistribution and targeting properties of ^64^Cu-CB-TE2A-G-ZHER2:342 and ^64^Cu-CB-TE2A-GEEE-ZHER2:342 were compared in tumor-bearing mice with the properties of ^64^Cu-NODAGA-ZHER2:S1, which had the best targeting properties in the previous study. ^64^Cu-CB-TE2A-GEEE-ZHER2:342 provided appreciably lower uptake in normal tissues and higher tumor-to-organ ratios than ^64^Cu-CB-TE2A-G-ZHER2:342 and ^64^Cu-NODAGA-ZHER2:S1. The most pronounced was a several-fold difference in the hepatic uptake. At the optimal time point, 6 h after injection, the tumor uptake of ^64^Cu-CB-TE2A-GEEE-ZHER2:342 was 16 ± 6%ID/g and tumor-to-blood ratio was 181 ± 52. In conclusion, a combination of the cross-bridged CB-TE2A chelator and Gly-Glu-Glu-Glu spacer is preferable for radiocopper labelling of Affibody molecules and, possibly, other scaffold proteins having high renal re-absorption.

## Introduction

A high level of human epidermal growth factor receptor type 2 (HER2) expression in breast and gastroesophageal cancer is a predictor for the response to HER2-targeting therapeutics (antibodies, antibody-drug conjugates, tyrosine kinase inhibitors)^1,2^. Several classes of radiolabelled probes for HER2 imaging (antibodies, antibody fragments, engineered scaffold proteins) are under development for non-invasive *in vivo* assessment of HER2 expression in disseminated cancer^3^. Affibody molecules are high-affinity binders engineered using a non-antibody protein scaffold. Due to their small size and high affinity, radiolabelled Affibody molecules can visualize different cancer-associated molecular target proteins with a high contrast just a few hours after injection^4^. Clinical data recently demonstrated that PET using the anti-HER2 ^68^Ga-ABY-025 Affibody molecule permits discrimination between breast cancer metastases with high and low levels of HER2 expression^5^. The largest difference between ^68^Ga-ABY-025 uptake in HER2-positive and HER2-negative metastases was obtained when images were acquired at 4 h after injection. We postulated that an extension of the time interval between injection and imaging would further increase the difference in uptake values in HER2-positive and negative metastases, thereby minimizing the risk of false-positive findings. Increasing the imaging time post-tracer injection requires a more long-lived positron-emitting radionuclide than ^68^Ga (T_1/2_ = 67.6 min).

The use of longer-lived positron-emitting copper isotopes, ^61^Cu (T_1/2_ = 3.4 h) or ^64^Cu (T_1/2_ = 12.7 h), may provide a broader time window for PET imaging of HER2 expression using Affibody molecules. However, besides selection of a nuclide with a suitable half-life and decay scheme, selection of an adequate labelling strategy is required. Since several studies suggested that the use of derivatives of the triazamacrocyclic chelator NOTA (2-[4,7-bis(carboxymethyl)-1,4,7-triazonan-1-yl]acetic acid) provided adequate *in vivo* stability of a radiocopper label^6–8^, we earlier evaluated the use of NOTA and NODAGA (2-(4,7-bis-carboxymethyl-1,4,7-triazonan-1-yl)-pentanedioic acid) for labelling of Affibody molecules with ^64^Cu^9^. In that study, the chelators were conjugated to the N-terminus of the synthetic Affibody molecule ZHER2:S1 via an amide bond. Both ^64^Cu-NOTA-ZHER2:S1 and ^64^Cu-NODAGA-ZHER2:S1 accumulated specifically in HER2-expressing xenografts. Surprisingly, the biodistribution pattern of both conjugates differed appreciably from that of other radiometal-labelled Affibody molecules. There was a rapid decrease of the renal radioactivity and an increase of radioactivity accumulation in healthy tissues with time, which resulted in a decrease of tumor-to-organ ratios. This effect was most pronounced for ^64^Cu-NOTA-ZHER2:S1. One explanation for such an observation was a release of radiometabolites from kidneys and their re-distribution. This effect was not observed in the  majority of earlier studies because the renal reabsorption of the tested tracer was much lower. This reasoning was corroborated by an observation of a similar effect for another polypeptide with a high renal reabsorption, A20FMDV2, which was labelled with ^64^Cu via NOTA monoamide^10^. Thus, a chelator with a higher metabolic stability or rapid excretion of radiometabolites is required for labelling of Affibody molecules or other proteins having high renal reabsorption with radiocopper.

**Figure 1:**
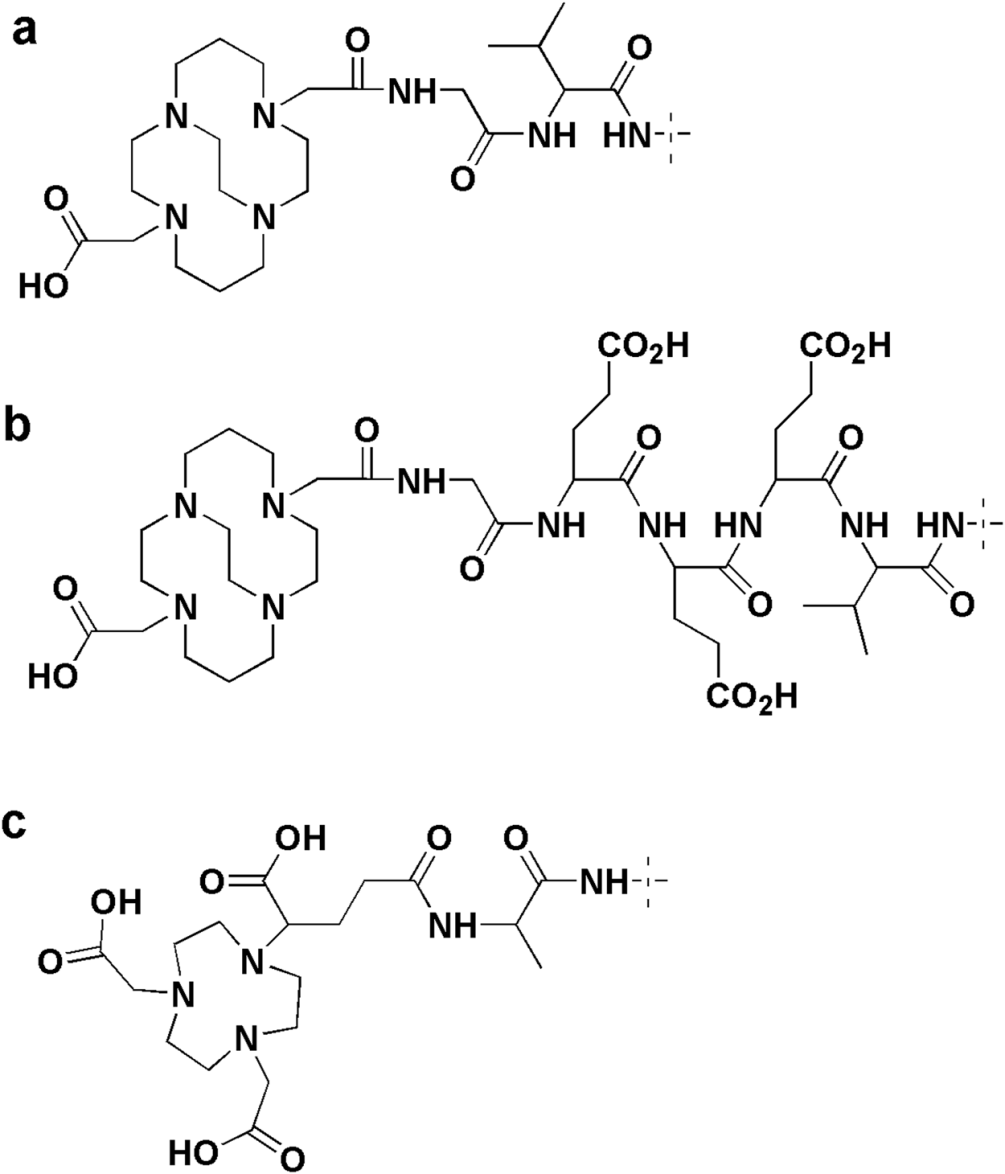
Structures of the chelators at the N-terminus of CB-TE2A-G-ZHER2:342 (**a**), CB-TE2A-GEEE-ZHER2:342 (**b**) and NODAGA-ZHER2:S1 (**c**).

The cross-bridged CB-TE2A (4,11-bis(carboxymethyl)-1,4,8,11-tetraazabicyclo[6.6.2]hexadecane) chelator provides a stable coupling of radiocopper to peptides and potentially meets our requirements^11,12^. However, previous studies have demonstrated that the use of chelators increasing the negative charge of the N-terminus of Affibody molecules is preferable due to reduced hepatic uptake^13–15^. The complex of Cu(II) with a monoamide derivative of CB-TE2A has a positive charge, which was considered as a potentially suboptimal property. Still, we have shown that the placement of a negatively charged triglutamyl spacer between the N-terminus of an Affibody molecule and a prosthetic group suppresses the hepatic uptake^16^. This was taken into account while designing the next generation of Affibody conjugates for labelling with radiocopper (Fig. 1). The aim of this study was to test the hypotheses that a) the use of monoamide CB-TE2A instead of monoamides of triaza chelators for radiocopper labelling of Affibody molecules prevents deterioration of tumor-to-organ ratios with time, and b) the use of a triglutamyl spacer between CB-TE2A and the N-terminus of the anti-HER2 Affibody molecule reduces undesirable hepatic uptake.

To test these hypotheses, we coupled CB-TE2A to the N-terminus of synthetic Affibody molecules extended either with a glycine (designation CB-TE2A-G-ZHER2:342) (Fig. 1a) or Gly-Glu-Glu-Glu spacer (CB-TE2A-GEEE-ZHER2:342) (Fig. 1b). The conjugates were labeled with ^64^Cu. Specificity of ^64^Cu-CB-TE2A-G-ZHER2:342 and ^64^Cu-CB-TE2A-GEEE-ZHER2:342 binding to HER2-expressing cells and their cellular processing after binding was evaluated using SKOV-3 ovarian carcinoma cell line. Biodistribution of ^64^Cu-CB-TE2A-G-ZHER2:342 and ^64^Cu-CB-TE2A-GEEE-ZHER2:342 was compared in BALB/C nu/nu mice bearing HER2-expressing SKOV-3 or HER2-negative Ramos lymphoma xenografts. The biodistribution was measured at 2, 6 and 24 h p.i. and compared with biodistribution of ^64^Cu-NODAGA-ZHER2:S1 (Fig. 1c), which had the best targeting properties in the previous study.

## Results

### Preparation and labeling of Affibody molecules

Affibody variants conjugated to the CB-TE2A chelator were successfully prepared by peptide synthesis and their identity was confirmed by mass spectrometry (Table 1). Purity of greater than 95% was obtained. Melting points (Table 1, Supplemental Figs S1 and S2) of both variants were close to the melting point of non-modified variant of ZHER2:342 (62.3 °C) and NODAGA-ZHER2:S1 (63 °C)^17^. The equilibrium dissociation constants for binding to HER2 were in close range (K_D_ = 60–90 pM) for all Affibody variants used in this study. CD spectra demonstrated high-fidelity refolding of CB-TE2A-containing Affibody molecules after thermal denaturation (Supplemental Figs S1 and S2).

**Table 1:** Properties of the tracers.

	Calculated MW, Da	Experimental MW, Da	KD, pM	Melting point, °C
CB-TE2A-G-ZHER2:342	7100	7099	60	63
CB-TE2A-GEEE-ZHER2:342	7487	7489	84	64
NODAGA-ZHER2:S1*	6977	6977	90	63

^*^Data from^17^.

Both CB-TE2A-G-ZHER2:342 and CB-TE2A-GEEE-ZHER2:342 were successfully labeled with ^64^Cu by incubation for 45 min at 95 °C in 0.55 M ammonium acetate, pH 5.6 followed by purification using NAP-5 size-exclusion columns. The isolated labelling yield was 84 ± 5% for ^64^Cu-CB-TE2A-G-ZHER2:342 and 88 ± 3% for ^64^Cu-CB-TE2A-GEEE-ZHER2:342. The radiochemical purity of the conjugates was over 98%. The maximum obtained specific radioactivity was 4.2 MBq/µg (29.6 GBq/µmol) and 3.9 MBq/µg (28.9 GBq/µmol) for ^64^Cu-CB-TE2A-G-ZHER2:342 and ^64^Cu-CB-TE2A-GEEE-ZHER2:342, respectively. Challenge with 500-fold excess of EDTA did not cause any detectable release of radionuclide from conjugates (Supplemental Table S2).

**Figure 2:**
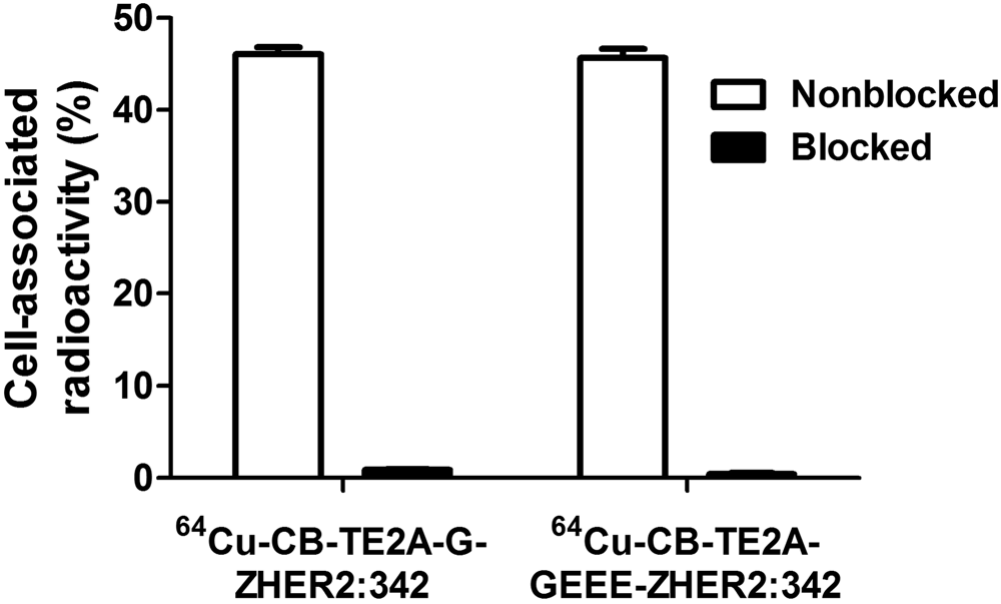
*In vitro* specificity of ^64^Cu-CB-TE2A-GEEE-ZHER2:342 and ^64^Cu-CB-TE2A-G-ZHER2:342 binding to HER2-expressing SKOV-3 cells. In blocked groups, receptors were pre-saturated by 200-fold excess of nonlabeled ZHER2:342. Data are presented as the mean of three samples and standard deviation.

**Figure 3:**
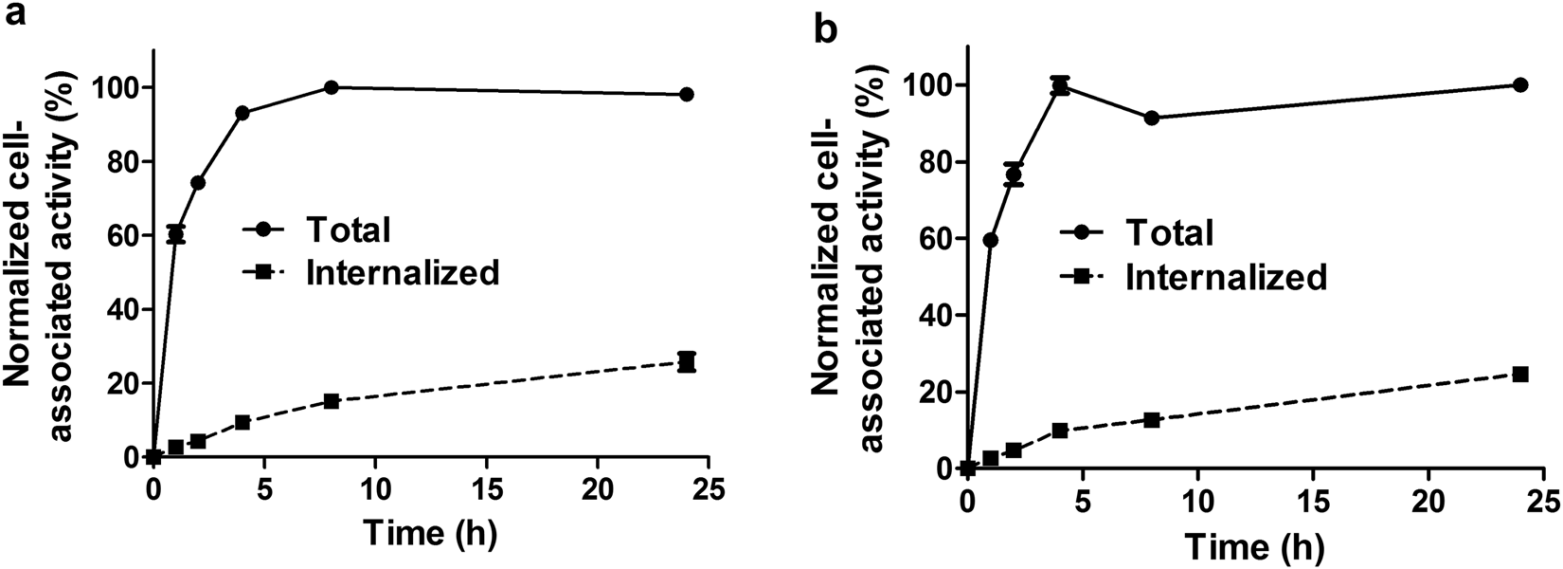
Cellular processing of ^64^Cu-CB-TE2A-GEEE-ZHER2:342 (**a**) and ^64^Cu-CB-TE2A-G-ZHER2:342 (**b**) during incubation with HER2-expressing SKOV-3 cells. Data are presented as the mean of three samples and standard deviation. Values are normalized to the maximum cell-associated radioactivity. Error bars might not be visible because they are smaller than symbols.

Binding of both ^64^Cu-CB-TE2A-G-ZHER2:342 and ^64^Cu-CB-TE2A-GEEE-ZHER2:342 to HER2-expressing SKOV-3 cells *in vitro* was efficiently prevented by pre-saturation of receptors by nonlabeled ZHER2:342 (Fig. 2), which demonstrated its HER2-specific nature. The internalization pattern (Fig. 3) was similar for both conjugates, and the internalized fraction at 24 h was approximately 25% for both variants. This was higher than the internalized fractions of ^64^Cu-NODAGA-ZHER2:S1 at this time point (6 ± 1%)^9^.

### Biodistribution Studies

**Figure 4:**
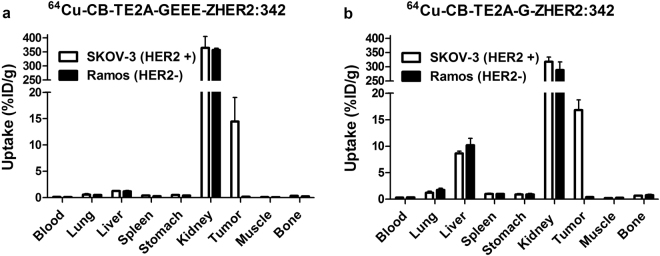
Specificity of HER2-targeting *in vivo*. Biodistribution of ^64^Cu-CB-TE2A-GEEE-ZHER2:342 (**a**) or ^64^Cu-CB-TE2A-G-ZHER2:342 (**b**) at 2 h p.i. in mice bearing either HER2-positive xenografts (SKOV-3) or HER2-negative xenografts (Ramos). The data are presented as mean ±SD for four mice.

To test HER2-specificity of ^64^Cu-CB-TE2A-GEEE-ZHER2:342 and ^64^Cu-CB-TE2A-G-ZHER2:342 accumulation in tumor *in vivo*, uptake of both tracers in HER2-positive (SKOV-3) and HER2-negative (Ramos lymphoma) xenografts was measured at 2 h post injection (p.i.). The results of the *in vivo* specificity tests are presented in Fig. 4. Uptake of ^64^Cu-CB-TE2A-GEEE-ZHER2:342 and ^64^Cu-CB-TE2A-G-ZHER2:342 in HER2-positive xenografts was significantly higher than in HER2-negative xenografts (p < 1 × 10^−4^). The non-specific uptake in Ramos xenografts was negligible, at 40-to-90-fold lower than the uptake in SKOV-3 xenografts.

Data comparing the biodistribution of ^64^Cu-CB-TE2A-G-ZHER2:342, ^64^Cu-CB-TE2A-GEEE-ZHER2:342 and ^64^Cu-NODAGA-ZHER2:S1 in mice bearing HER2-positive SKOV-3 xenografts are presented in Table 2. At 2 h after injection, the tumor uptake for all three conjugates was in the range of 14–17% ID/g. There was no significant difference between tumor uptake of the different conjugates at any of time points. Furthermore, there was no significant difference between tumor uptake at different time points for each conjugate. All variants demonstrated initially (2 h p.i.) a biodistribution pattern typical for Affibody molecules, i.e. clearance from the majority organs and tissues (although to different extent) and a high renal re-absorption of the tracers. Low initial radioactivity in intestines with content indicated negligible contribution of hepatobiliary excretion to clearance. The biodistribution pattern of ^64^Cu-NODAGA-ZHER2:S1 was in a good agreement with the data published earlier^9^, i.e. relatively rapid decrease of radioactivity from kidneys (3-fold between 2 and 24 h) and gradual increase of radioactivity uptake in other normal tissues over time. The biodistribution pattern of ^64^Cu-CB-TE2A-G-ZHER2:342 and ^64^Cu-CB-TE2A-GEEE-ZHER2:342 was more typical for other radiometal-labeled Affibody molecules. Uptake in normal organs and tissues decreased over time for conjugates containing cross-bridged chelators. It should be noted that the uptake of CB-TE2A-conjugated variants was lower in the majority of normal organs compared with ^64^Cu-NODAGA-ZHER2:S1 at 2 h p.i. Renal radioactivity cleared more slowly compared with ^64^Cu-NODAGA-ZHER2:S1, at approximately 2-fold between 2 and 24 h after injection. However, there was a clear difference between ^64^Cu-CB-TE2A-G-ZHER2:342 and ^64^Cu-CB-TE2A-GEEE-ZHER2:342. The most prominent was the difference in hepatic uptake, which was 5–7-fold lower for ^64^Cu-CB-TE2A-GEEE-ZHER2:342. In addition, the uptake of ^64^Cu-CB-TE2A-GEEE-ZHER2:342 in lung, spleen, muscle and bone was 1.5–2.5-fold lower.

**Table 2:** Biodistribution of ^64^Cu-CB-TE2A-G-ZHER2:342, ^64^Cu-CB-TE2A-GEEE-ZHER2:342 and ^64^Cu-NODAGA-ZHER2:S1 in mice bearing HER2-positive (SKOV-3) xenografts. Data are presented as mean ± SD for four mice.

	Uptake, % ID/g								
	2 h			6 h			24 h		
	^64^Cu-CB-TE2A-GEEE-ZHER2:342	^64^Cu-CB-TE2A-G-ZHER2:342	^64^Cu-NODAGA-ZHER2:S1	^64^Cu-CB-TE2A-GEEE-ZHER2:342	^64^Cu-CB-TE2A-G-ZHER2:342	^64^Cu-NODAGA-ZHER2:S1	^64^Cu-CB-TE2A-GEEE-ZHER2:342	^64^Cu-CB-TE2A-G-ZHER2:342	^64^Cu-NODAGA-ZHER2:S1
Blood	0.15 ± 0.03	0.29 ± 0.08	0.34 ± 0.08^c^	0.09 ± 0.01	0.11 ± 0.01^b^	0.43 ± 0.04^c^	0.07 ± 0.01	0.10 ± 0.01^b^	0.73 ± 0.04^c^
Lung	0.6 ± 0.2^a^	1.2 ± 0.3^b^	1.8 ± 0.2^c^	0.42 ± 0.03^a^	0.70 ± 0.04^b^	2.3 ± 0.4^c^	0.34 ± 0.05^a^	0.61 ± 0.08^b^	3.6 ± 0.2^c^
Liver	1.2 ± 0.1^a^	8.7 ± 0.4^b^	5.2 ± 0.7^c^	1.2 ± 0.1^a^	6.7 ± 0.8^b^	5.8 ± 0.4^c^	0.77 ± 0.07^a^	5.1 ± 0.2^b^	6.9 ± 0.6^c^
Spleen	0.40 ± 0.04^a^	1.0 ± 0.1	0.8 ± 0.2^c^	0.37 ± 0.02^a^	0.78 ± 0.02^b^	0.96 ± 0.08^c^	0.28 ± 0.02^a^	0.60 ± 0.09^b^	1.6 ± 0.2^c^
Stomach	0.51 ± 0.06^a^	0.9 ± 0.1	1.8 ± 0.2^c^	0.38 ± 0.02	0.57 ± 0.03^b^	2.0 ± 0.2^c^	0.22 ± 0.04	0.5 ± 0.2^b^	2.3 ± 0.3^c^
Kidney	364 ± 41	318 ± 17	349 ± 14	272 ± 18	252 ± 14	295 ± 43	185 ± 15^a^	141 ± 11^b^	111 ± 20^c^
Tumor	14 ± 5	17 ± 2	14 ± 5	16 ± 6	14 ± 3	12 ± 5	11 ± 3	14 ± 4	16 ± 5
Muscle	0.12 ± 0.01^a^	0.20 ± 0.03^b^	0.25 ± 0.02^c^	0.09 ± 0.01	0.13 ± 0.02^b^	0.27 ± 0.02^c^	0.06 ± 0.01	0.10 ± 0.01^b^	0.38 ± 0.04^c^
Bone	0.32 ± 0.09^a^	0.63 ± 0.06	0.5 ± 0.1^c^	0.19 ± 0.03	0.4 ± 0.1	0.6 ± 0.1^c^	0.14 ± 0.06^a^	0.39 ± 0.08^b^	0.8 ± 0.1^c^
Intestines*	1.1 ± 0.2^a^	1.7 ± 0.1^b^	3.5 ± 0.4^c^	0.8 ± 0.1	1.5 ± 0.1	5.3 ± 0.5^c^	0.3 ± 0.1^a^	1.1 ± 0.4^b^	5.6 ± 0.4^c^

^*^Data for intestines with content are presented as %ID per whole sample.

^a^Significant difference (p < 0.05) between ^64^Cu-CB-TE2A-GEEE-ZHER2:342 and ^64^Cu-CB-TE2A-G-ZHER2:342 at this time point.

^b^Significant difference (p < 0.05) between ^64^Cu-CB-TE2A-G-ZHER2:342 and ^64^Cu-NODAGA-ZHER2:S1 at this time point.

^c^Significant difference (p < 0.05) between ^64^Cu-CB-TE2A-GEEE-ZHER2:342 ^64^Cu-NODAGA-ZHER2:S1 at this time point.

The biodistribution features of ^64^Cu-CB-TE2A-G-ZHER2:342, ^64^Cu-CB-TE2A-GEEE-ZHER2:342 and ^64^Cu-NODAGA-ZHER2:S1 had a clear influence on their tumor-to-organ ratios (Table 3). ^64^Cu-NODAGA-ZHER2:S1 demonstrated the lowest values at 2 h after injection, and the tumor-to-blood, tumor-to-lung, and tumor-to-spleen ratios decreased over time. ^64^Cu-CB-TE2A-GEEE-ZHER2:342 demonstrated the best ratios at all time points. Particularly important was the highest tumor-to-liver ratio. At 6 h after injection, tumor-to-blood and tumor-to-bone ratios for ^64^Cu-CB-TE2A-G-ZHER2:342 were significantly higher than at 2 h (p < 0.05), however there was no significant increase of any ratios from 6 to 24 h.

**Table 3:** Comparison of ^64^Cu-CB-TE2A-G-ZHER2:342, ^64^Cu-CB-TE2A-GEEE-ZHER2:342 and ^64^Cu-NODAGA-ZHER2:S1 tumor-to-organ ratios in nude mice bearing SKOV-3 xenografts. Data are presented as mean ± SD for four mice.

	Tumor-to-organ ratio								
	2 h			6 h			24 h		
	^64^Cu-CB-TE2A-GEEE-ZHER2:342	^64^Cu-CB-TE2A-G-ZHER2:342	^64^Cu-NODAGA-ZHER2:S1	^64^Cu-CB-TE2A-GEEE-ZHER2:342	^64^Cu-CB-TE2A-G-ZHER2:342	^64^Cu-NODAGA-ZHER2:S1	^64^Cu-CB-TE2A-GEEE-ZHER2:342	^64^Cu-CB-TE2A-G-ZHER2:342	^64^Cu-NODAGA-ZHER2:S1
Blood	95 ± 21	61 ± 18	41 ± 8^c^	185 ± 66	134 ± 36^b^	29 ± 13^c^	162 ± 39	140 ± 53^b^	22 ± 7^c^
Lung	25 ± 6^a^	14 ± 4	8 ± 2^c^	39 ± 14^a^	20 ± 6^b^	6 ± 3^c^	34 ± 8	23 ± 7^b^	4 ± 1^c^
Liver	11 ± 3^a^	1.9 ± 0.2	3 ± 1^c^	13 ± 4^a^	2.2 ± 0.7	2.1 ± 0.9^c^	15 ± 3^a^	2.7 ± 0.8	2.3 ± 0.7^c^
Spleen	35 ± 8^a^	17 ± 3	18 ± 4^c^	44 ± 14^a^	18 ± 4	13 ± 5^c^	40 ± 12	24 ± 10	10 ± 3^c^
Stomach	28 ± 7	19 ± 4^b^	8 ± 2^c^	42 ± 13	25 ± 7^b^	6 ± 3^c^	53 ± 15	30 ± 15^b^	7 ± 2^c^
Kidney	0.04 ± 0.01	0.05 ± 0.01	0.04 ± 0.01	0.06 ± 0.02	0.06 ± 0.01	0.04 ± 0.02	0.06 ± 0.01	0.10 ± 0.03	0.14 ± 0.04^c^
Muscle	116 ± 35	86 ± 16	57 ± 19^c^	181 ± 52	118 ± 43^b^	47 ± 20^c^	194 ± 72	149 ± 59	41 ± 14^c^
Bone	46 ± 12^a^	27 ± 2	25 ± 5	87 ± 30^a^	38 ± 11	22 ± 6^c^	81 ± 7^a^	36 ± 4^b^	20 ± 8^c^

^a^Significant difference (p < 0.05) between ^64^Cu-CB-TE2A-GEEE-ZHER2:342 and ^64^Cu-CB-TE2A-G-ZHER2:342 at this time point.

^b^Significant difference (p < 0.05) between ^64^Cu-CB-TE2A-G-ZHER2:342 and ^64^Cu-NODAGA-ZHER2:S1 at this time point.

^c^Significant difference (p < 0.05) between ^64^Cu-CB-TE2A-GEEE-ZHER2:342 and ^64^Cu-NODAGA-ZHER2:S1 at this time point.

### *in vivo* Imaging Studies

**Figure 5:**
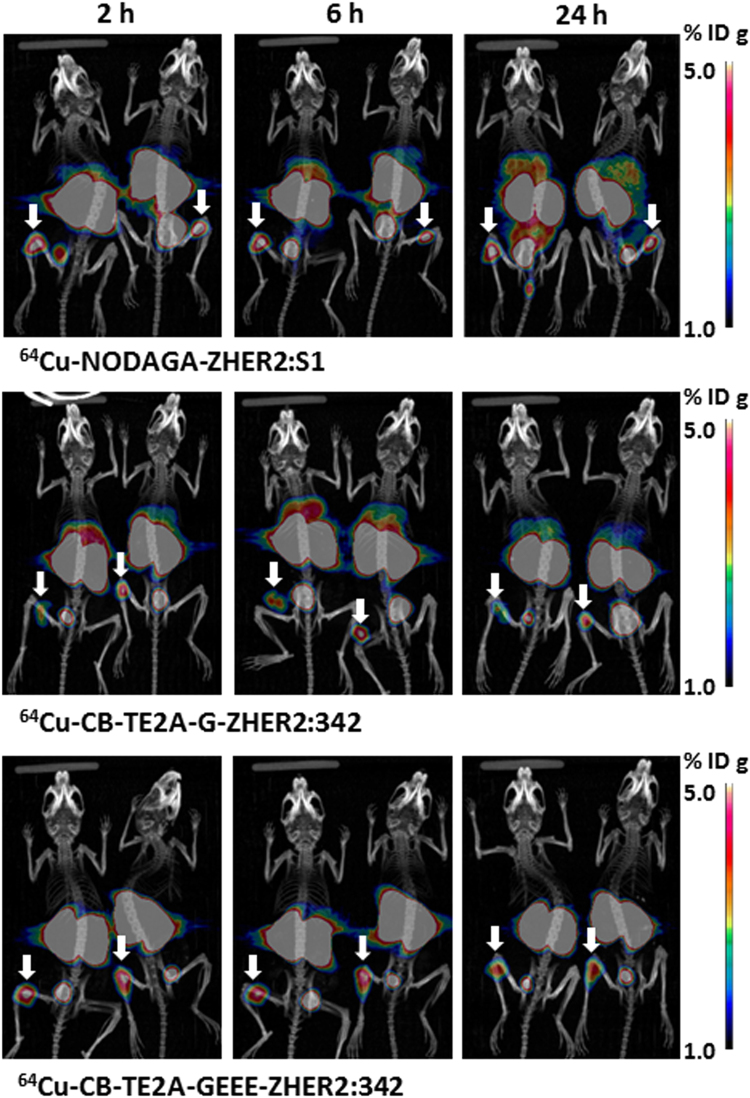
Maximum-intensity-projection PET/CT images of ^64^Cu-NODAGA-ZHER2:S1, ^64^Cu-CB-TE2A-G-ZHER2:342 and ^64^Cu-CB-TE2A-GEEE-ZHER2:342 at 2, 6, and 24 h after injection in mice bearing HER2-positive SKOV-3 xenografts. Arrows point at tumors. The scale was adjusted to provide clear visualization of hepatic uptake of ^64^Cu-CB-TE2A-G-ZHER2:342342 and ^64^Cu-NODAGA-ZHER2:S1. This resulted in saturation of tumor images (white spots).

PET/CT imaging was performed at the same time point as measurements of biodistribution to verify the data and obtain a visual confirmation. The PET imaging results (Fig. 5) confirmed the *ex vivo* measurement data. Tumors were clearly visualized by all radiolabelled Affibody molecules. It has to be noted that the xenografts were small, which might result in an underestimation of the uptake in some tumors due to partial volume effect^18^. Indeed, the tumor weights in this study were ranging from 12 to 68 mg. Assuming a spherical shape for these tumors their diameters ranged approximately from 2.5 mm to 5 mm. The activity recovery coefficient (RC) of a PET scanner is dependent on the size of the object imaged and the energy of the β^+^ particles emitted by a particular radionuclide. Previous investigations^19,20^ demonstrated that the Inveon PET provides the RC for ^18^F varying from 0.15 for objects with 1 mm diameter to 0.97 for 5 mm objects. ^64^Cu and ^18^F have similar β^+^ energies and one can expect a similarly low recovery for small tumors also for ^64^Cu. Besides tumors, very prominent radioactivity uptake was observed for all variants in kidneys and occasionally in bladder. In addition, high liver uptake was observed for ^64^Cu-NODAGA-ZHER2:S1 and ^64^Cu-CB-TE2A-G-ZHER2:342 and accumulation of radioactivity in intestines was visualized for ^64^Cu-NODAGA-ZHER2:S1 at 6 and 24 h p.i. ^64^Cu-CB-TE2A-GEEE-ZHER2:342 provided the best contrast for imaging of HER2-positive xenografts.

## Discussion

Numerous studies have demonstrated that CB-TE2A is one of the best chelators for labelling of peptides with ^64^Cu^12^. The main limitation of its use is the requirement of high temperature for complexation of copper^21^, which complicates its use for labelling of proteins with a tertiary structure, e.g. monoclonal antibodies, due to the risk of denaturation. This is not an issue for Affibody molecules, which are capable of rapid refolding under physiological conditions^22^. Although a chelator could interfere with this process, the CD spectra measurements demonstrated that both CB-TE2A-G-ZHER2:342 and CB-TE2A-GEEE-ZHER2:342 refold with high fidelity after thermal denaturation (Supplemental Figs S1 and S2). After labelling at 95 °C, both ^64^Cu-CB-TE2A-GEEE-ZHER2:342 and ^64^Cu-CB-TE2A-G-ZHER2:342 retained the capacity of binding specifically to HER2-expressing cells both *in vitro* and *in vivo* (Figs 2 and 4). Conjugation of CB-TE2A and the amino acid linkers had essentially no effect on the affinity of the construct, which was in the range of 60–90 pM (Table 1).

The main finding of this study is that the use of CB-TE2A for labelling of Affibody dramatically changes the pattern of uptake in normal organs compared with the use of NODAGA (Table 2). While the uptake of radioactivity in blood, liver, lung, spleen, stomach, muscles and bones demonstrated an ascending trend for ^64^Cu-NODAGA-ZHER2:S1, the trend was clearly descending for both variants labelled using CB-TE2A. This resulted in significantly increased tumor-to-organ ratios with time. The effect might be partially explained by better retention of renal catabolites of ^64^Cu-CB-TE2A-GEEE-ZHER2:342 and ^64^Cu-CB-TE2A-G-ZHER2:342 compared to the retention of ^64^Cu-NODAGA-ZHER2:S1 radiometabolites, as the decrease of renal radioactivity over time is more pronounced for ^64^Cu-NODAGA-ZHER2:S1. This is in agreement with the data provided by Fani and co-workers^8^ for ^64^Cu-labed somatostatin antagonists showing higher retention of radioactivity kidneys when CB-TE2A was used instead of NODAGA. It has to be noted that that absolute renal uptake of somatostatin analogues was much lower compared to Affibody molecules, and re-distribution of radiometabolites had no obvious effect of uptake in normal tissues. It is also possible that the radiometabolites are excreted more rapidly in the case of cross-bridged chelators. The high kinetic stability of the ^64^Cu-CB-TE2A chelate compared to ^64^Cu-NODAGA may also account for increased clearance of the CB-TE2A-Affibody constructs from organs other than the kidneys.

Another essential finding is the role of a linker between a chelate and the N-terminus of Affibody molecules. Incorporation of a chelator/radiometal complex alters the charge of an imaging probe both locally and globally and affects off-target interactions and consequently on normal tissue uptake. Earlier study suggested that a negative charge of a radionuclide-chelator complex at the N-terminus of an anti-HER2 Affibody molecule is preferable^13–15^ and a positively charged monoamide-CB-TE2A/Cu(II) complex might slow down the blood clearance and increase the hepatic uptake. In fact, the liver uptake of ^64^Cu-CB-TE2A-G-ZHER2:342 was approximately four-fold higher than the uptake of the same molecules labelled with ^111^In using DOTA^23^. The present study demonstrated that this effect can be counteracted by the incorporation of a negatively charged triglutamyl linker. The hepatic uptake of ^64^Cu-CB-TE2A-GEEE-ZHER2:342 was 5-6-fold lower than the uptake of ^64^Cu-CB-TE2A-G-ZHER2:342 (Table 2), which translated into substantially higher tumor-to-liver ratios (Table 3). This is a critical aspect since both breast and esophageal cancers often have liver metastases^24^.

It has to be noted that there may be an alternative approach to avoiding the positive charge of the radiocopper-CB-TE2A complex at N-terminus of affibody molecules. Synthesis of CB-TE2A derivatives featuring an additional orthogonally protected arm for conjugation has been reported^25,26^. Such derivatives would provide a neutral complex with Cu^2+^. However, the use of oligoglutamate linker provides more straightforward and flexible way for modification of N-terminal charge for affibody molecules.

Overall, ^64^Cu-CB-TE2A-GEEE-ZHER2:342 provides the best tumor-to-organ ratios among the tested variants. Biodistribution data suggest that imaging at 6 or 24 h after injection would improve the tumor-to-blood ratios and therefore increase the sensitivity of imaging. Better retention of Affibody molecules in tumors with high expression, which was observed both in preclinical^27^ and clinical studies^5^, should ensure reliable discrimination between metastases with high and low HER2 expression at later time points.

Recent clinical studies demonstrated that the elevated renal uptake of Affibody molecules^5^ or nanobodies^28^ does not complicate PET imaging of metastases in the lumbar area and is associated with an acceptable level of renal and whole body absorbed doses. This provides a rationale for the use of scaffold protein-based imaging probes with high renal uptake, such as DARPins^29^, fibronectin domain derivatives^30^, or ADAPTs^31^. Some of them, e.g. EGFR-targeting Affibody molecules^32^, would have increased sensitivity of imaging when used several hours or at the next day after injection. We hope that the results of this study would help to choose the optimal labeling approach for such imaging agents. Of course, a clinical translation would require a careful optimization of dosimetry for each imaging agent. For example, data from this study suggest that the optimum time point for imaging would be around 6 h after injection, since there is no significant increase of tumor-to-organ ratios after this. This indicates that ^61^Cu (T_1/2_ = 3.4 h) might be a preferable copper radioisotope for labeling of anti-HER2 Affibody molecules. This radionuclide might be produced by the ^60^Ni(d,n)^61^Cu or ^61^Ni(p,n) ^61^Cu nuclear reactions using low-energy cyclotrons available at PET centers^33,34^. Preliminary estimations suggest that the renal and whole body doses would be similar in this cases to doses due to imaging using ^68^Ga-labeled Affibody molecules.

In conclusion, this study confirmed our hypotheses that the use of monoamide CB-TE2A instead of monoamino derivatives of triaza chelators for radiocopper labelling of Affibody molecules prevents deterioration of tumor-to-organ ratios with time, and the use of a triglutamyl spacer between CB-TE2A and the N-terminus of the anti-HER2 Affibody molecule reduces undesirable hepatic uptake. The combination of CB-TE2A and a negatively charged Gly-Glu-Glu-Glu linker is a suitable variant for labelling of Affibody molecules with radiocopper.

## Methods

### Radioactivity measurements, analysis and data treatment

VDC-405 ionization chamber (Veenstra Instruments) was used for radioactivity measurement during formulation of solutions for injections. Radioactivity of samples from *in vitro* and *in vivo* studies was measured using an automated gamma spectrometer (Wizard^2^, PerkinElmer). Purity, yield and stability of radiolabelled Affibody molecules were measured using radio-instant thin-layer chromatography (radioITLC) with ITLC-SG strips (Agilent). The strips were developed with 0.2 M citric acid, pH 2. Radioactivity distribution along the strips was measured using BAS-TR2025 imaging plates (Fuji Photo Film Co.) scanned with an FLA-5100 scanner (both from Fuji Photo Film Co). RadioITLC analysis was validated using a blank experiment and radio-reversed phase high performance liquid chromatography (radio-HPLC). For radio-HPLC, a Jupiter Proteo C12 column (4.6 × 250 mm) was eluted with a linear CH_3_CN/H_2_O gradient (10–70% CH_3_CN in 12 min) in 0.1% trifluoroacetic acid at a flow rate of 1.0 mL/min.

The data are presented as the mean +/− standard deviation. An unpaired two-tailed t-test was used to find a significant difference (P < 0.05) between two sets of data. One-way ANOVA analysis with Bonferroni’s multiple comparison test using Prism 5 software (GraphPad Software) was used to evaluate differences between more than two sets.

### Synthesis and characterization of Affibody molecules

The chelator CB-TE2A was synthesized as previously described^11^.

The Affibody molecule ZHER2:342 with the sequence VENKFNKEMRNAYWEIALL PNLNNQQKRAFIRSLYDDPSQSANLLAEAKKLNDAQAPK was synthesized using standard Fmoc chemistry on a 433A Peptide Synthesizer (Applied Biosystems) as described earlier^17^. The N-terminus of the synthesized Affibody molecule was extended manually by coupling of Gly (variant G-ZHER2:342) or Gly-Glu-Glu-Glu sequence (variant GEEE-ZHER2:342). The CB-TE2A chelator (10 mol equivalents) was conjugated to the peptide using 10 mol equivalents of N′,N′-dicyclohexylcarbodiimide and N-ethyldiisopropylamine. The molar excess of the chelator was used to ensure nearly 100% conjugation yield. Deprotection of the protecting groups and release of the peptide from the resin were achieved by treatment with a mixture of H_2_O/TIS/TFA (2.5:2.5:95) for 2 h at RT. The deprotected peptide was extracted between H_2_O and tert-butyl methyl ether, followed by lyophilization of the aqueous phase. Analysis of the conjugates was performed by RP-HPLC using a SOURCE^TM^ 5RPC column, a 20 min gradient of 20–50% B (A: 0.1%TFA-H_2_O, B: 0.1%TFA-CH_3_CN) and a flow rate of 1 mL/min. The purification was performed using a Zorbax CB300-C18 column. Mass spectrometric analysis was carried out on a Bruker MALDI mass spectrometer (CCA matrix) to confirm the protein mass.

The affinity of CB-TE2A-GEEE-ZHER2:342 and CB-TE2A-G-ZHER2:342 binding to the extracellular domain of HER2 (R&D systems) was measured in duplicate with concentrations of 0.25, 0.5, 1, 2, 3, 5, and 10 nM using Biacore 2000 as described earlier^17^. The curve fitting (Langmuir 1:1 binding model) was performed using BiaEval software (Biacore AB, Sweden).

Circular dichroism studies were performed with 40 µM protein in PBS (pH 7.4) using a Jasco J-810 spectropolarimeter^17^. For the variable temperature measurements, the signal was recorded at 221 nm and the temperature was increased from 20 to 90 °C at a rate of 5 °C/min. Circular dichroism spectra were recorded from 250 to 195 nm at 20 °C before and after the melting.

The Affibody molecule NODAGA-ZHER2:S1 containing the NODAGA chelator conjugated via amide bond at the N-terminus was synthesized, purified and characterized as described earlier^17^. This Affibody molecule has a sequence AEAKYAKEMRNAYWEIALLPNL TNQQKRAFIRKLYDDPSQSSELLSEAKKLNDSQAPK, and biodistribution of ^68^Ga- and ^111^In-labeled DOTA conjugated ZHER2:S1and ZHER2:342 are very similar^35,36^.

Copper-64 was produced using the ^64^Ni(p,n)^64^Cu nuclear reaction and separated and purified by anion exchange chromatography, as previously described^37^. The radionuclide was prepared as a solution in 0.04 M HCl with effective specific radioactivity of 3 TBq/μmol at the end of bombardment.

For radiolabeling, Affibody molecule, 50 µg in 50 µL 0.55 M ammonium acetate, pH 5.6, was mixed with 150–270 MBq ^64^CuCl_2_ in 10 µL 0.04 M HCl, and the mixture was incubated for 45 min at 95 °C. A 500-fold excess of Na_4_EDTA (10 mg/mL in water, 137 µL) was added to the mixture followed by additional incubation at 95 °C for 10 min. The radiolabeled Affibody molecules were purified using disposable NAP-5 size-exclusion columns (GE Healthcare). The label stability was tested by incubation of labeled Affibody molecules with a 500-fold excess of Na_4_EDTA during 1 h.

NODAGA-ZHER2:S1 was labeled with ^64^Cu for comparative *in vivo* studies as described earlier^9^. As HER2-postitive cells, SKOV-3 ovarian carcinoma cell line (1.2 × 10^6^ receptors per cell)^22^ was used for *in vitro* and *in vivo* studies. Ramos lymphoma cell line was used for implantation of HER2-negative xenografts. Both cell lines were purchased from American Type Tissue Culture Collection (ATCC) via LGC Standards (Teddington, UK) and cultivated according to ATCC recommendations.

The specificity of ^64^Cu-CB-TE2A-GEEE-ZHER2:342 and ^64^Cu-CB-TE2A-G-ZHER2:342 binding to HER2-expressing SKOV-3 cells was evaluated by a saturation assay using a 200-fold molar excess of non-labelled ZHER2:342 Affibody molecules^9^.

To evaluate internalization, SKOV-3 cells were incubated with the conjugates (1 nM) and the internalized radioactivity was measured at 1, 2, 4, 8 and 24 h using a modified acid wash method, as described earlier^38^. The cells were incubated at 37 °C with the labeled compounds (concentration 1 nM). A set of three dished was used for each data point. The incubation medium from a dishes was removed. The cells were washed ice cold medium. The membrane-bound radioactivity was removed by treatment with 0.5 mL of 0.2 M glycine buffer containing 4 M urea, pH 2.0, for 5 min on ice. Dishes were additionally washed with 0.5 mL acidic buffer and by 1 mL PBS, and the fractions were pooled. To collect radioactivity internalized by the cells, the cells were treated them with 0.5 mL of 1 M NaOH at 37 °C for 0.5 h. The dishes were additionally washed with 0.5 mL NaOH solution followed by 1 mL PBS. The radioactivity in acidic (cell bound) and alkaline (internalized) fraction was measured.

### Biodistribution Studies

Animals were cared for in compliance with the guidelines of the International Council of Laboratory Animal Science. All animal procedures were approved by the Animal Ethics Committee of the Provincial Government of Southern Finland, and performed following the guidelines of the European Community Council Directives 86/609/EEC.

HER2-positive xenografts were obtained by subcutaneous inoculation of 1 × 10^7^ SKOV-3 cells in female BALB/C nu/nu mice (Scanbur, 8 weeks old at arrival). HER2-negative control tumors were obtained by inoculation of 1 × 10^7^ Ramos lymphoma cells. Experiments were performed 20 days after implantation. The average tumor weight was 38 ± 22 mg at the time of the experiment.

A group of four mice per data point was used for *ex vivo* measurements. Mice bearing SKOV-3 xenografts were injected via the tail vein with ^64^Cu-CB-TE2A-G-ZHER2:342, ^64^Cu-CB-TE2A-GEEE-ZHER2:342 or ^64^Cu-NODAGA-ZHER2:S1 (450 kBq in 100 µL per mouse). The injected protein dose was adjusted to 5 µg (0.7 nmol). Biodistribution was measured 2, 6, and 24 h post-injection. Mice bearing HER2-negative Ramos lymphoma xenografts were injected with the same amounts of protein and radioactivity, and biodistribution was measured 2 h after injection. The animals were exsanguinated under anesthesia, the organs and tissues of interest were excised and their weight and radioactivity were measured. Radioactivity of gastrointestinal tract (with content) was also measured to evaluate hepatobiliary excretion.

### *in vivo* Imaging Studies

SKOV-3 (*n* = 2) xenograft-bearing mice were injected via the tail vein with ^64^Cu-CB-TE2A-G-ZHER2:342 (13.1 ± 0.9 MBq, 5 µg, 100 µL). ^64^Cu-CB-TE2A-GEEE-ZHER2:342 (16.6 ± 0.05 MBq, 5 µg, 100 µL) or ^64^Cu-NODAGA-ZHER2:S1 (12.3 ± 0.9 MBq, 5 µg, 100 µL). Mice were anesthetized using 2.5% isoflurane/O_2_, and positioned on a heating pad two at a time in a PET/CT scanner (Siemens Medical Solutions, Inc.) for CT acquisition (10 min) and PET scan in list mode (20 min). Mice were scanned at 2, 6, and 24 h post-injection. PET images were reconstructed using an FBP algorithm of two iterations, followed by maximum *a posteriori* (MAP, 18 iterations) integrative algorithms (Inveon Acquisition Workplace, version 2.0; Siemens Preclinical Solutions). Data were decay-corrected to the time of injection.

## Electronic supplementary material

Supporting Information
