# Supplementary material for: Molecular design of radiocopper-labelled Affibody molecules
Source: Sci Rep. 2018 Apr 25;8:6542. doi: 10.1038/s41598-018-24785-2 (PMC5916907; doi:10.1038/s41598-018-24785-2)
Supplement: Supplementary file 1 — Supporting Information [file 41598_2018_24785_MOESM1_ESM.pdf]

SUPPLEMENTARY INFORMATION

Molecular design of radiocopper-labelled affibody molecules

Vladimir Tolmachev[1, ☺,] Tove J Grönroos [2, 3, 4, ☺], Cheng-Bin Yim [2, 5,], Javad Garousi[1], Ying Yue[6], Sebastian Grimm[6], Johan Rajander [5], Anna Perols [6], Merja Haaparanta-Solin [2, 7], Olof Solin [2, 5, 7] , Riccardo Ferdani[8], Anna Orlova [9], Carolyn Anderson [10], Amelie Eriksson Karlström[6].

[☺] VT and TJG contributed equally

[1] Department of Immunology, Genetics and Pathology, Uppsala University, Uppsala, Sweden;
[2] Turku PET Centre, University of Turku, Turku, Finland;
[3] MediCity Research Laboratory, University of Turku, Turku, Finland;

[4] Department of Oncology and Radiotherapy, Turku University Hospital, Turku, Finland

[5] Turku PET Centre, Åbo Akademi University, Turku, Finland;
[6]Division of Protein Technology, School of Biotechnology, KTH Royal Institute of Technology, Stockholm, Sweden;

[7] Department of Chemistry, University of Turku, Turku, Finland;
[8] Washington University, St. Louis, MO
[9] Department of Medicinal Chemistry, Uppsala University, Uppsala, Sweden
[10] Departments of Medicine, Radiology, Bioengineering and Pharmacology & Chemical Biology, University of Pittsburgh, Pittsburgh, PA, 15203, USA

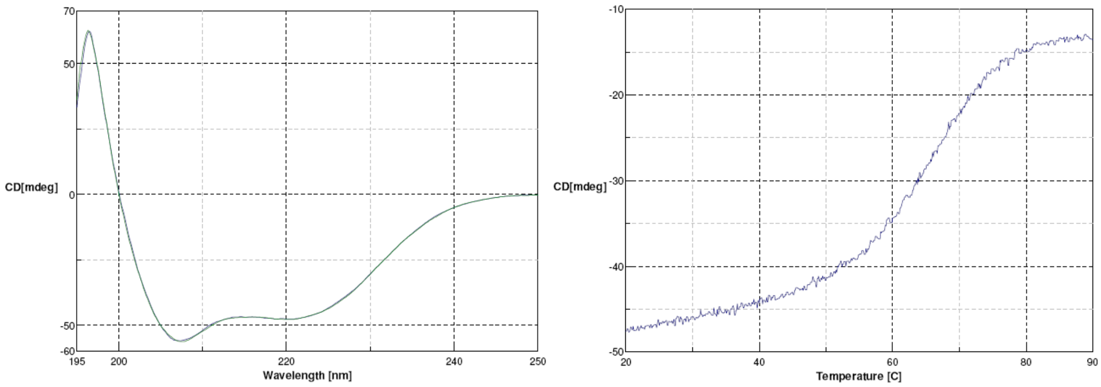

Figure S1. CD studies of CB–TE2A–GEEE–ZHER2:342. CD spectra recorded at 20 °C before and after variable temperature measurements (left) and the thermal melting curve recorded at 221 nm (right).

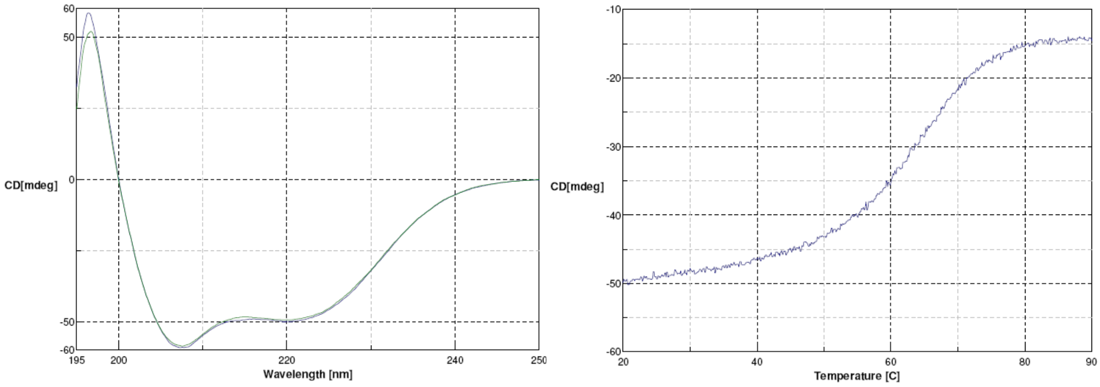

Figure S2. CD studies of CB–TE2A–G–ZHER2:342. CD spectra recorded at 20°C before and after variable temperature measurements (left) and the thermal melting curve recorded at 221 nm (right)

Table S1. Stability of ^{64}Cu-CB-TE2A-GEEE-ZHER2:342 and ^{64}Cu-CB-TE2A-G-ZHER2:342 under 1-h challenge with 500-fold excess of EDTA.

	Peptide-associated radioactivity (%)	
	^{64}Cu-CB-TE2A-G-ZHER2:342	^{64}Cu-CB-TE2A-GEEE-ZHER2:342
EDTA solution	99.1 ± 0.0	99.8 ± 0.3
PBS (control)	99.3 ± 0.2	99.7 ± 0.1
